# Introduction of a new repair technique in bony avulsion of the FDP tendon: A biomechanical study

**DOI:** 10.1038/s41598-018-28250-y

**Published:** 2018-07-02

**Authors:** Gabriel Halát, Lukas L. Negrin, Ewald Unger, Thomas Koch, Johannes Streicher, Jochen Erhart, Patrick Platzer, Stefan Hajdu

**Affiliations:** 1 Department for Orthopaedics and Trauma Surgery, Medical University of Vienna, Vienna, Austria; 2 Center for Medical Physics and Biomedical Engineering, Medical University of Vienna, Vienna, Austria; 3 Institute of Materials Science and Technology, Faculty of Mechanical and Industrial Engineering, TU Wien, Vienna, Austria; 4 Department for Anatomy and Biomechanics, Karl Landsteiner University of Health Sciences, Krems an der Donau, Austria

## Abstract

The purpose of this study was to determine the biomechanical characteristics of an innovative surgical technique based on a tension banding principle using a suture anchor in the repair of bony avulsions of the flexor digitorum profundus tendon. After injury simulation in 45 fresh frozen distal phalanges from human cadavers, repair was performed with minifragment screws, interosseous sutures and the innovative technique (15 per group). All repairs were loaded for a total of 500 cycles. Subsequently the specimens were loaded to failure. Load at failure, load at first noteworthy displacement (>2 mm), elongation of the system, gap formation at the avulsion site, and the mechanism of failure were assessed. The new techniques’ superior performance in load at failure (mean: 100.5 N), load at first noteworthy displacement (mean 77.4 N), and gap formation (median 0 mm) was statistically significant, which implies a preferable rigidity of the repair. No implant extrusion or suture rupture during cyclic loading were recorded when the new technique was applied. This innovative repair technique is superior biomechanically to other commonly used surgical tendon reattachment methods, particularly with respect to an early passive mobilisation protocol. Further, due to its subcutaneous position, reduction of complications may be achieved.

## Introduction

Surgical approach to a bony avulsion of the flexor digitorum profundus (FDP) tendon is challenging due to a variety of avulsion fragment size, fragment displacement and the scarce soft tissue covering the injury and the repair.

Leddy and Packer (1977) described three types of FDP tendon avulsion injury^1^. Type III is defined by a bony avulsion of the FDP tendon where the bony fragment remains distal to the A4 pulley^1–4^. A severe fragment displacement (>2 mm) requiring surgical repair is common, as the mechanism of the injury is a forced hyperextension of the distal interphalangeal (DIP) joint while the finger is being flexed actively. In the last decades, a notable variety of surgical repair techniques has been published, some of them craving for a decrease in intraoperative and postoperative complications, in some cases with varying success^1,3,5–11^. To achieve a good functional and aesthetic result after the repair of a bony avulsion of the FDP tendon, accurate reduction, rigidity of the repair, as well as an adequate postoperative mobilisation protocol focusing on the balance between protection of the repair and prevention of scar contracture formation are required^5,12,13^. In general, treatment with minifragment screws, interosseous sutures, pullout button or miniplate is favourized. Still there are dozens of other repair techniques, in prevalence interosseous refixation techniques and implant combinations, described^6^. Extracutaneous repair constructs hold a relevant complication potential^3,7,8,14^. To avoid infections and repair component rupture due to manipulation, subcutaneous repair techniques, such as screw repair and interosseous sutures and/or wiring are currently in use. However, when addressing small fragmentary avulsions, a risk of implant cut-out or disintegration of the bony fragment when being drilled and tightened has been observed. This may result in recurrence of the avulsion and/or loss of joint congruity, which contributes to subsequent loss of range of motion (ROM) and posttraumatic arthrosis at the DIP Joint.

Until now, suture anchors gained acceptance in the field of surgical tendon and ligament repair, and with respect to FDP avulsions in particular, reattachment of the tendon in the absence of bony avulsion. Believing in the potential of suture anchors being used in the repair of bony FDP avulsions as well, we designed an interosseous suture technique based on a tension banding principle using a suture anchor. We expect to avoid a relevant range of reported complications as well as to address small fragmentary bony avulsions.

The aim of this study was to introduce the new repair technique and to evaluate its biomechanical characteristics, expecting an increase of rigidity and stability during an early mobilisation protocol. Furthermore, we compared clinically relevant biomechanical parameters of our new technique to minifragment screw repair and interosseous sutures, the two nowadays most frequently used subcutaneous surgical repair techniques for type III FDP tendon avulsions.

## Material and Methods

Considering numerous methodological findings in the literature, we utilized an experimental design that examines cyclic loading and load to failure in a curvilinear testing model with the aim to evaluate techniques for reconstruction of flexor tendon injuries in zone I^9,10,15^. We applied this design to investigate the biomechanical characteristics of the innovative repair technique compared to interosseous sutures and screw repair. None of the tissues used in this study were procured from prisoners. All specimens were obtained at the Center for Anatomy and Cell Biology, Medical University of Vienna where all of the participants provided written informed consent. The Institutional Ethics Committee of the Medical University of Vienna approved this study. All methods were performed in accordance with the relevant guidelines and regulations.

### Specimen preparation

Twenty-three cadaveric specimens (14 female, 9 male) were randomly selected from the same age category (mean 75, range 65–85 years of age). None of these presented with a record of increased loss of bone density due to particular diseases, as well as during preparation, none exhibited any signs of impaired bone quality. Forty-five nonrandomized, fresh-frozen distal phalanges with an intact FDP tendon attachment from the ring, middle, and index fingers were harvested. During preparation we exarticulated each distal phalanx at the DIP joint, preserving only the FDP tendon attached (FDP tendon length: approx. 10 cm). The cross-sectional area (CSA) of the tendon was determined at the level of the A4 pulley by diameter measurement and calculation of a circle area. Soft tissue surrounding the tendon insertion to the distal phalanx, the volar plate and the A5 pulley were excised to expose the repair construct and to allow visual evaluation of the repair site. At the volar aspect of the distal phalanx, a bony fragment including 30% of the articular line with an intact FDP tendon insertion was carefully removed from the distal phalanx using a chisel.

Saline solution (0.9% NaCl) was used to rehydrate the specimens upon testing and to irrigate them periodically throughout preparation and testing.

**Figure 1:**
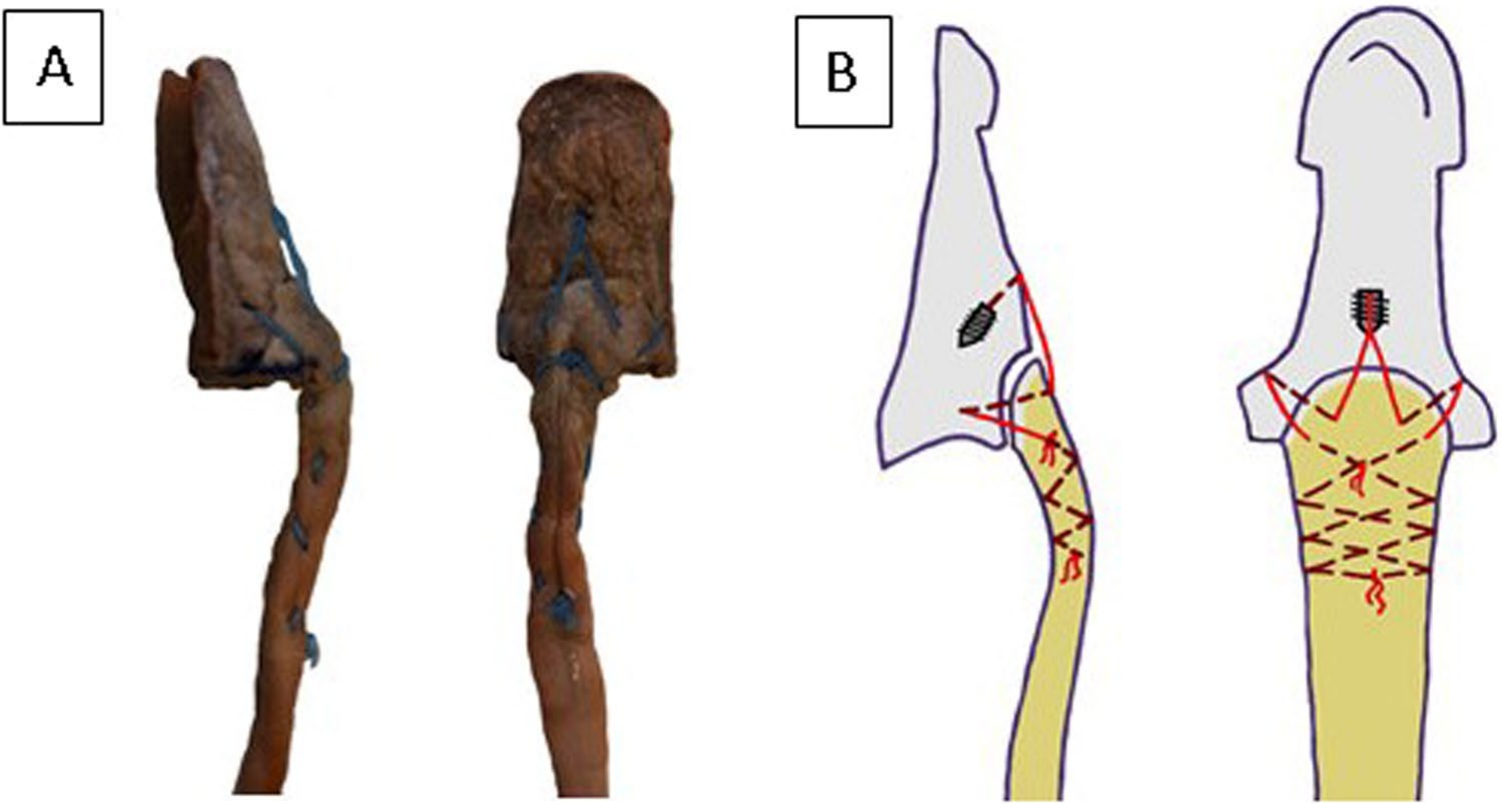
(**A**) Suture insertion into the avulsed fragment and securing the tendon with the Bunnell technique in a cadaver specimen. (**B**) Graphical illustration of the new repair technique.

By random allocation, three study groups of 15 specimens each were formed. In the first group (termed new), the bony tendon avulsion repair was performed with our innovative technique using a Corkscrew 2.2 × 4 mm suture anchor loaded with a #2-0 FiberWire (Arthrex, Naples, Florida) (Fig. 1). The mean CSA of the FDP tendon was 9.4 ± 2.1 mm^2^. We regard intact cortical bone distal to the distal margin of the tendon avulsion site as an appropriate suture anchor insertion point. In accordance with the deadman theory of suture anchors we chose a retrograde insertion angle of 45°^7,16,17^. Following the principle of tension banding, we performed an interosseous attachment of the bony fragment with the two strands of the anchor suture. After avulsion fragment reduction, each suture was inserted into the bony fragment at the borders of the median to the lateral thirds of the volar surface of the distal phalanx. The sutures were then guided out at the lateral intact corticalis of the distal phalanx and firmly tightened. Both sutures were then placed underneath the tendon at the level of the volar base of the distal phalanx, perforating the tendon in the median third and were tightly woven together. The procedure was finalized with a Bunnell suture grasping the tendon 1 cm proximally and being secured with a 5-throw square knot^18^ (Fig. 1).

**Figure 2:**
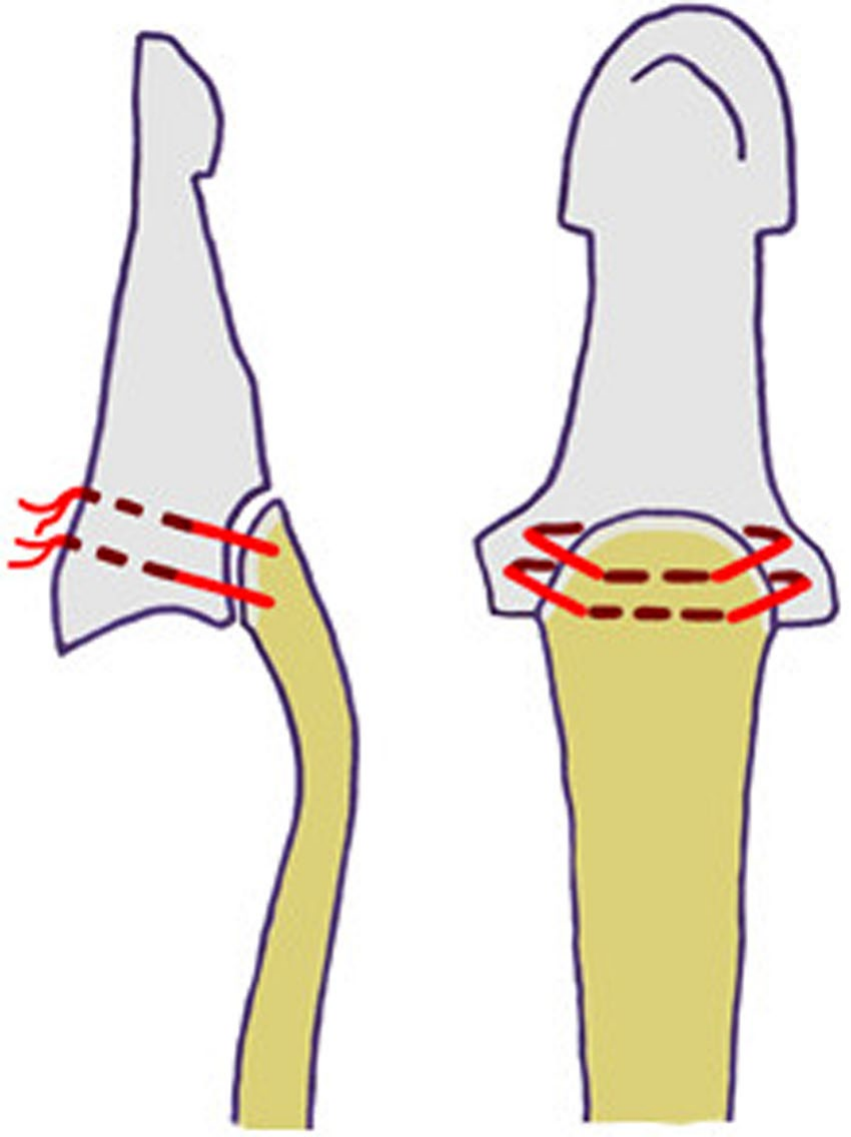
Illustration of the interosseous sutures to reattach a bony avulsion of the FDP tendon.

In the second group (termed interosseous), the bony avulsion was repaired with interosseous sutures as described by Moiemen and Elliot^11^. Similar to the suture material mounted in the suture anchor in the first group (new), a #2-0 FiberWire suture (Arthrex, Naples, Florida) was used (Fig. 2). Calculation of the CSA in this group resulted in a mean of 9.0 ± 2.1 mm^2^.

**Figure 3:**
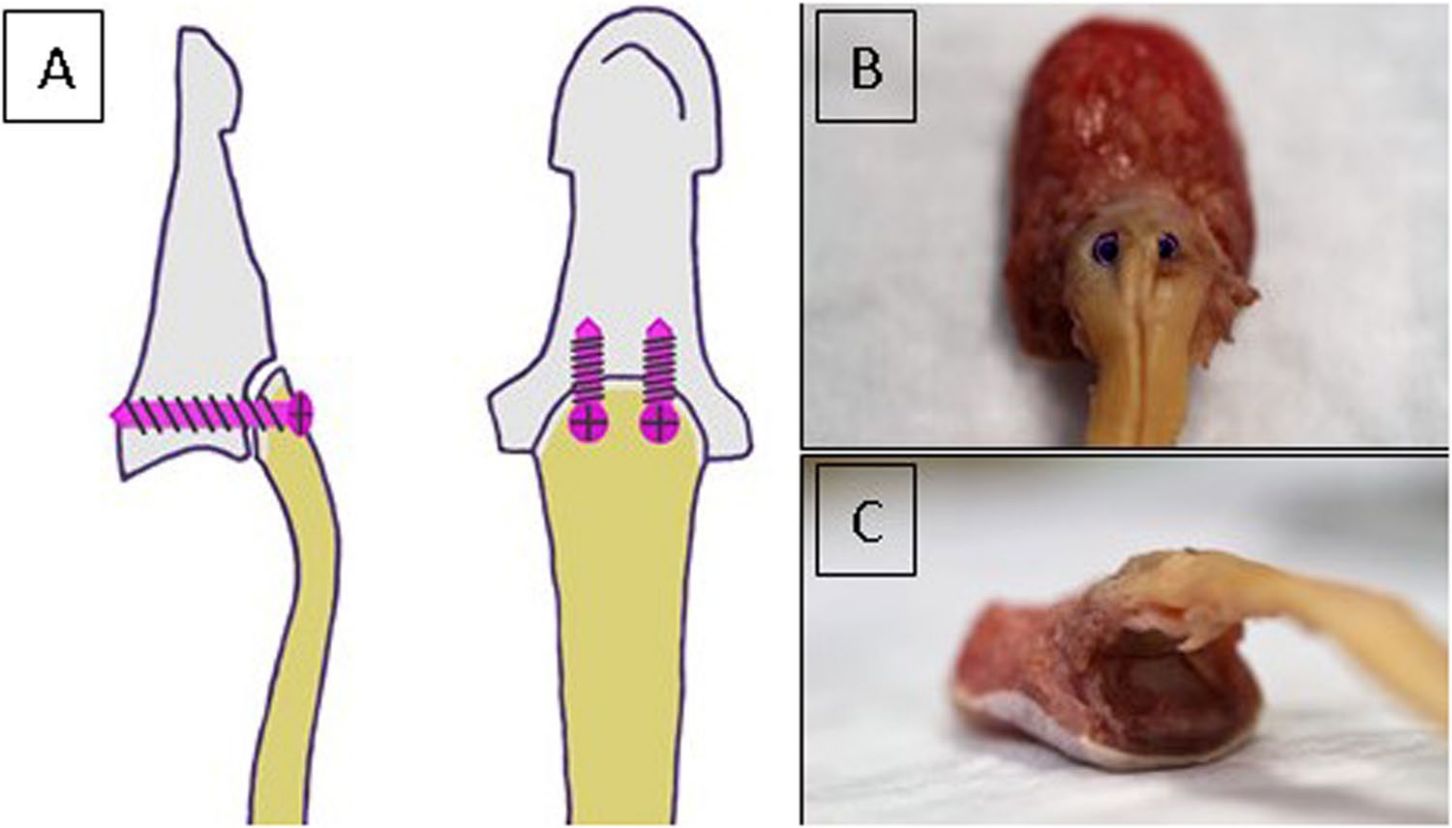
(**A**) Bony tendon avulsion repair using minifragment screws. (**B**) Screw positioning in a cadaver specimen. (**C**) Reduced articular line of the distal phalanx after repair with minifragment screws.

In the third group (termed screw) bony FDP tendon avulsions were kept in reduced position by two cannulated cortical titanium screws 1.8 × 6 mm (Medberg Solutions, Anthering, Austria). The mean CSA of the tendons repaired with screws was 10.1 ± 1.6 mm^2^. The screws were inserted over two guidewires after verification of an acceptable reduction and an aligned articular line (Fig. 3). Special attention was given to an adequate hold of the screws to avoid early extrusion during testing.

### Testing and data collection

**Figure 4:**
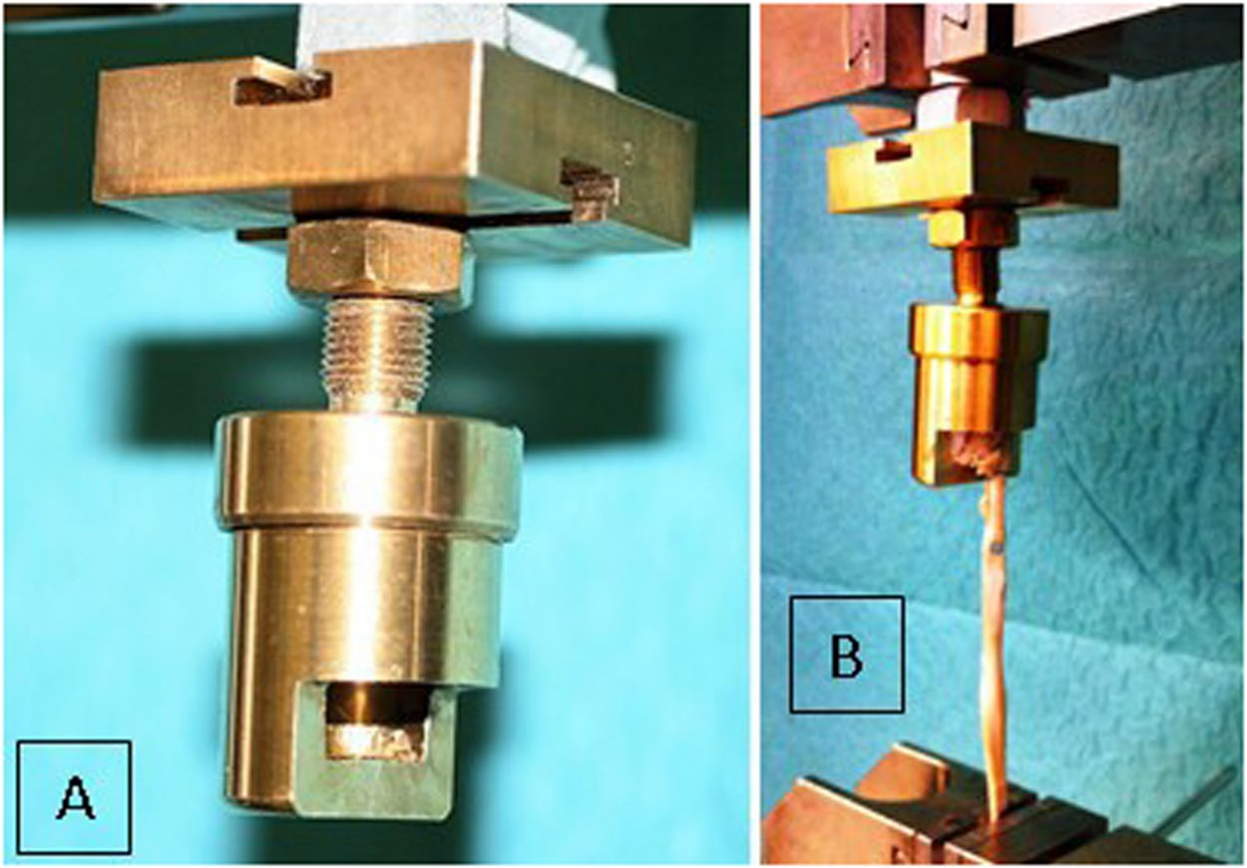
(**A**) Specimen holding device with a semicircumferrential window. The ball-joint is situated at the top of the cylinder. (**B**) Testing unit with the DP cylinder. The tendon is secured in the inferior clamp using abrasive paper to prevent tendon slipping.

**Figure 5:**
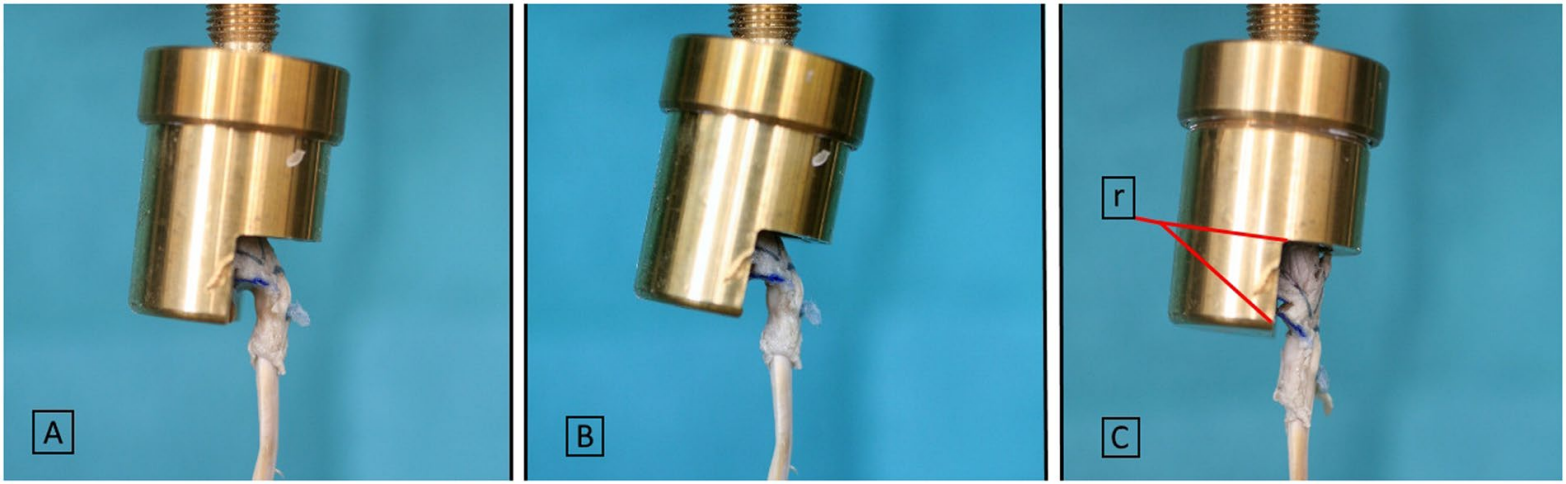
A specimen repaired with the new technique (**A**) prior to cyclic loading, (**B**) after 500 cycles, and when loaded to failure with a displacement of 3 mm (**C**). Distance (r) is the main length reference and measures exactly 10 mm.

At completion of repair, the specimens were mounted in a distal phalanx (DP) cylindrical holding device. We designed this device with a semicircumferrential window (Fig. 4) to allow full view of the repair site, leaving all repair components unaffected. We used no implants or other invasive techniques to keep the distal phalanx stable inside the cylinder. To prevent specimen tilt or rotation inside the cylinder throughout testing, it was centered using common plaster. Alignment of the bone–tendon complex along the longitudinal axis of the distal phalanx and thus prevention of impingement of structures at the repair site was ensured by a ball-joint on the top of the cylinder. The base of the distal phalanx, as well as the base of the reduced avulsion fragment with an intact insertion of the FDP tendon were marked to visualize repair site dynamics during cyclic loading and load to failure in the most consistent manner. This construct was mounted on an electro-mechanical material tensile testing machine (Zwick Z050, Zwick GmbH, Ulm, Germany) equipped with a 1 kN load cell (Fig. 4). The accuracy of the load cell in the low load region was checked by calibrated weights, and the maximum deviation was 7 mN in the range from 0.1 to 5 N. A standard tensile clamp was used to retain the proximal end of the tendon 7 cm proximal to the repair. This distance was consistent throughout all evaluations, thus enabling accurate data on elongation of the whole specimen– suture complex. Testing was conducted under a preload of 2 N. Continuous cyclic loading was applied to the specimens from 2 to 15 N at a rate of 5 N/s for a total of 500 cycles without interruption. A photograph was taken with a high-resolution camera placed in front of the experimental setup to measure gap formation at the attachment site at the initial 2 N preload, after every 100 cycles, as well as during loading to failure to monitor any bias affecting the reliability of the measured data. The lateral aspect of the reduced articular line was labelled with a blue felt tip pen allowing gap formation observation throughout testing (Fig. 5). Subsequently, specimens were loaded to failure at 20 mm/min upon completion of the cyclic testing. Claiming that load at failure will not represent the load at the occurrence of a significant articular step-off, load at first noteworthy displacement (>2 mm) was separately evaluated to present a clinically relevant failure of the repair. In addition, the following parameters were recorded and analysed as well: elongation of the system, gap formation (displacement of the fragment, thus resulting in an articular line step off), load at failure, and the mechanism of failure. We evaluated elongation between the 50th and the 500th cycle to avoid bias given by individual tendon morphology as the system should reach full structural balance after 50 cycles. Displacement of the bony fragment documented on the photographs was analysed with ImageJ Software (National Institutes of Health, Washington, DC, United States).

### Statistical analysis

Statistical analysis was conducted using IBM SPSS Statistics Version 23, 64-bit. Normal distribution was assessed by the Kolmogorov-Smirnov-Test. To determine any differences in the properties of the investigated three repair techniques we performed one-way analysis of variance (ANOVA) followed by Tukey’s method as a post hoc test for pairwise comparison in case of normal distribution. For non-normally distributed samples the Kruskal-Wallis test and Dunn’s post hoc test were used. Normally distributed parameters are presented as mean and standard deviation, whereas parameters of skew distributions are displayed as median and interquartile range in round brackets. P-values lower than 0.05 were considered statistically significant.

## Results

**Figure 6:**
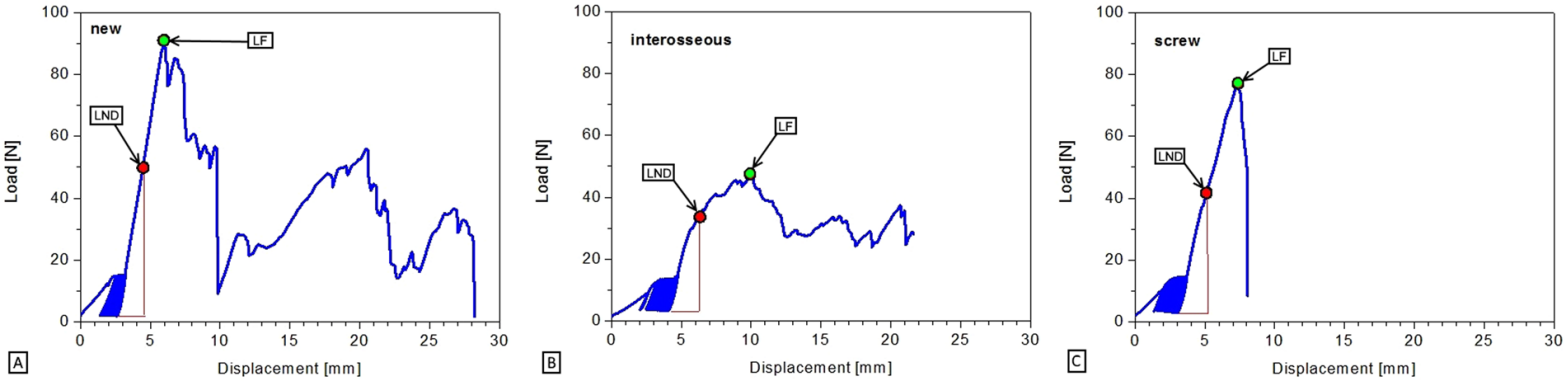
Graphs (**A**–**C**) are representing exemplary load versus displacement curves for each particular repair method. Abb. LND indicates the point of measurement of the load at the first noteworthy displacement (>2 mm). Abb. LF marks the point of measurement of the load at failure.

**Figure 7:**
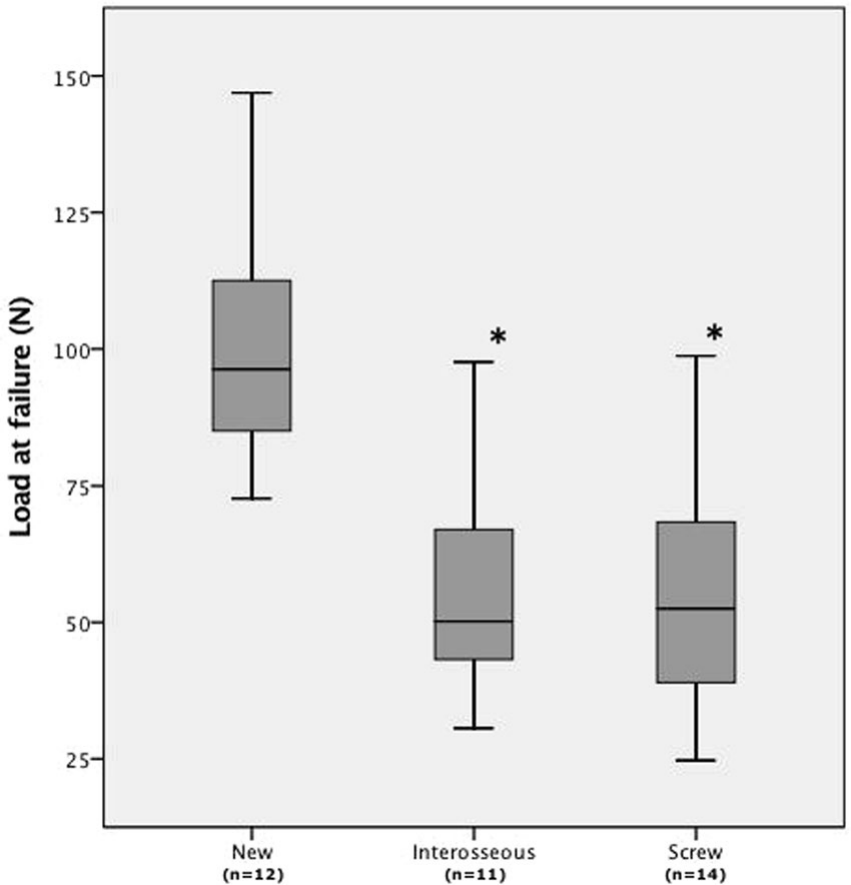
Boxplots of load at failure data. *Statistically significant difference compared to our novel technique.

We encountered no difficulties performing the selected repair procedures. The repairs showed no structural failures during cyclic loading such as premature extrusion of the implants, anchor or suture extraction, screw breakage, suture rupture, tendon rupture, or technical failure of the testing machine. No statistically significant difference with respect to the CSA of the FDP tendons was detected between the three study groups. To visualize the range of elongation and the course of the force throughout the complete loading and extrusion process a displacement versus force curve for each tendon repair was generated (Fig. 6). Comparing the elongation of the repair complex, no statistically significant superiority (p = 0.124) of either implant was found. Between the 50th and the 500th cycle, we observed a mean elongation of 0.3 ± 0.2 mm in the new technique group, 0.9 ± 0.4 mm in the interosseous group and 0.4 ± 0.2 mm in the specimens repaired with screws. Upon 500 cycles, evaluation of data from photographic images revealed, that gap formation at the repair site was significantly greater using the interosseous sutures or screws for repair compared with the new technique. Median gap formation after cyclic loading was 0 mm (IQR 0) in the new repair technique group compared to 0.5 mm (IQR 0–2) in the group repaired by interosseous sutures (p = 0.006) and compared to 0 mm (IQR 0–2.5) in the screw repair group (p = 0.044). Difference in gap formation between the groups interosseous suture repair and screw repair was statistically not significant (p = 0.705). After cyclic loading, the repairs were loaded to failure. Evaluating the load at first noteworthy displacement (>2 mm), we observed a statistically significant superiority of the new repair technique (p < 0.0001) compared to the two other repairs (Table 1). When loaded to failure, suture anchors failed either by suture/anchor pullout from the bone or extrusion of the distal phalanx from the specimen holding cylinder. Specimens repaired with interosseous sutures failed by suture rupture in most cases. Prior to failure, a point of inflection was usually seen as a sign of breakage of a suture strand. When screws were used as the method of repair, we observed a failure by either screw pullout, or extrusion of the distal phalanx from the cylinder (Table 2). Specimens, in which the distal phalanx was pulled out from the cylinder, were excluded from the load at failure measurement in all three study groups, as these do not represent a failure of the repair. Disintegration of repairs in the final stage of continuous loading to failure occurred at lower loads in the interosseous repair (mean 56.5 ± 20.4 N) and screw repair (mean 53.5 ± 21.2 N) groups compared to new repair technique group (mean 100.5 ± 21.0 N), showing an inferiority in load tolerance. This was statistically significant (p < 0.0001) (Fig. 7).

**Table 1:** Tension forces of the repair techniques at the first noteworthy displacement.

	New (n = 15)	Interosseous (n = 15)	Screw (n = 15)
Load at first noteworth. displ. (N) mean ± SD	77.4 ± 25.8	39.8 ± 17.1*	48.0 ± 19.2*

*Statistically significant difference compared to our novel technique.

**Table 2:** Failure mechanisms of the repair techniques at uninterrupted increasing load.

	New (n = 15)	Interosseous (n = 15)	Screw (n = 15)
Pullout of specimen from the cylinder	3	4	1
Suture/Anchor pullout	12	—	—
Screw failure/pullout	—	—	14
Suture rupture	—	11	—

## Discussion

Repair of bony FDP tendon avulsions holds a significant risk of peri- and postoperative complications. The aim of this study was to address some of these complications by developing an innovative repair technique and testing its biomechanical characteristics in an early passive mobilisation protocol simulation. Observing the clinical aspects of FDP tendon repairs so far, infections due to penetrating implant parts, lesions to the nail matrix with persisting nail deformities, skin necrosis and scar strictures resulting in extension deficit in the DIP joint were reported^3,7–10,14^. These may originate from the approach itself, or may be triggered by the implant and/or technique used for repair. Intentions to reduce implant load at a repair site with a scarce soft tissue layer, to prevent secondary implant removal surgery and opposing skin penetrating repair techniques were the leading thoughts when designing the new repair technique. To decrease heterogeneity within the study groups, we chose to examine subcutaneous repairs only. Designing the first comparative group, we referred to the study of Moiemen and Elliot (2000) investigating interosseous wires in type III FDP tendon injuries^11^. Eglseder and Russell first described a type IV injury repair using a single cortical minifragment screw in 1990^19^. Proposing an improved rotational stability and hold, we selected a double screw repair technique for the second comparative group. In the present literature, there have been no reports of suture anchor application in the repair of bony avulsions of the FDP tendon, probably due to the expectation of low rigidity and insufficient hold. Nevertheless, in addition to the advantages of avoiding an external repair construct, suture anchors placed into the distal phalanx also allow the surgeon to use a biomechanically superior locking suture^6,7,9,10,20,21^. Choosing a single Corkscrew suture anchor in particular, is based on the findings of a study performed by our research group in 2014, where we were able to demonstrate the superior biomechanical characteristics and the reliability of this implant^21^. In the present study, the resistance of a single suture anchor to high physiological loads was appropriate for a passive mobilisation protocol, and performed superior to both other used techniques, even when considering the double anchor repair proposed by Burstein *et al*.^9^. When solid fixation cannot be achieved, recurrence of the avulsion and/or loss of joint congruity may be persistent and may contribute to subsequent loss of range of motion and posttraumatic arthrosis at the DIP Joint. Gap formation leading to an articular line step off and subsequently bearing the potential of arthrosis was significantly lower in repairs using the new technique. In clinical setting, failure of the repair complex can occur as a result of anchor-bone fixation failure, or suture breakout from the anchor, bone or soft tissue^11,20–31^. However, we never observed this kind of failure during cyclic loading of the repair.

Passive mobilisation after FDP tendon repair is imperative to prevent ROM impairment due to scar formation, confronting the repair construct with higher demands on structural integrity. We adopted a testing protocol designed by Latendresse *et al*., loading the repaired specimen in a cyclic manner to simulate postoperative passive mobilization, enabling us to observe gap formation and load associated behaviour of the repair^15^. We defined failure to be clinically relevant at a fragment displacement of more than 2 mm, thus introducing the load at the first noteworthy displacement (>2 mm) parameter into our biomechanical protocol. Our results in repair complex elongation may be interpreted as a proof of rigidity of the new repair technique being similar to the elongation parameters of the screw repair. Reflecting the description of Leversedge and colleagues (2002) about nutritive supply of flexor tendon insertions we advocate, that immediate repair is possible but not urgently required due to an adequate vascularisation of the bony fragment by the intact vincula of the FDP tendon^32^.

The *ex vivo* design of this biomechanically complex scientific inquiry is a relevant limitation. Future biomechanical investigations, with the complete flexor tendon sheath preserved, will be necessary to point out the dynamic behaviour of the here evaluated FDP tendon avulsion repair technique. Moreover, examinations under clinical conditions will be essential to confirm the herein demonstrated *ex vivo*, biomechanical superiority of the new technique compared to two other commonly used repair procedures, as well as to disclose potential complications which cannot be anticipated in a biomechanical study.

## Conclusion

We were able to demonstrate the superior biomechanical characteristics of the here proposed new repair technique compared to two other commonly used techniques for the repair of a bony FDP tendon avulsion. Although we focused on biomechanics of repair techniques only, our findings indicate a potential improvement with respect to peri- and postoperative complications. Clinical investigations will be needed to confirm a potential *in vivo* improvement in the therapeutic approach to these injuries.
